# Comparison between objective and subjective postoperative intraocular pressure immediately after cataract surgery

**DOI:** 10.5935/0004-2749.2023-0174

**Published:** 2024-10-31

**Authors:** Julia F. Heringer, Gustavo Rosa Gameiro, Maria Fernanda Abalem, Pedro C. Carricondo

**Affiliations:** 1 Department of Ophthalmology, Hospital das Clínicas, Faculdade de Medicina, Universidade de São Paulo, São Paulo, SP, Brazil; 2 Department of Ophthalmology and Visual Sciences, Escola Paulista de Medicina, Universidade Federal de São Paulo, São Paulo, SP, Brazil; 3 Bascom Palmer Eye Institute, University of Miami Miller School of Medicine, University of Miami, Miami, FL, USA; 4 Department of Ophthalmology and Visual Sciences, University of Michigan Medical School, Ann Arbor, MI, USA

**Keywords:** Cataract, Intraocular pressure, Hypotony, Tonometry, Eye diseases, Training

## Abstract

**Purpose:**

To compare objective and subjective intraocular pressure measurements immediately after cataract surgery and intraocular pressure measurements between less experienced surgeons (Group 1) and experienced surgeons (Group 2).

**Methods:**

Surgeons were asked to estimate the IOP after corneal sealing after surgery based on their tactile perception of eye tension (subjective intraocular pressure) Objective intraocular pressure was measured using a Perkins tonometer while patients were still in the surgical field. Objective intraocular pressure was compared to subjective intraocular pressure. Results from less experienced surgeons were compared to more experienced surgeons.

**Results:**

The study comprised 81 surgeries (81 eyes) performed by 27 surgeons. The mean objective intraocular pressure (9.14 mmHg; SD=5.86) was statistically significantly lower (p<0.001) than the mean subjective intraocular pressure (19.21 mmHg; SD=4.82). Hypotony (intraocular pressure <6mmHg) was observed in 25 eyes (30.86%). The mean subjective intraocular pressure was 18.8 mmHg (SD=5.19) for less experienced surgeons and 19.5 mmHg (SD=4.46) for more experienced, without statistically significant difference (p=0.541). No statistically significant difference (p=0.71) was observed when comparing objective intraocular pressure in Group 1 (10.32 mmHg; SD=6.65) and Group 2 (7.97 mmHg; SD=4.7).

**Conclusion:**

Objective intraocular pressure was significantly lower than subjective intraocular pressure, regardless of surgeons’ experience. This study showed that the subjective method is unreliable compared to the gold standard (Perkins tonometer) and does not improve with surgeons’ experience. Establishing standard training methods is paramount to developing surgeons’ skills.

## INTRODUCTION

The successful completion of every surgical step during phacoemulsification (phaco) is paramount for an optimal surgical outcome. The final step of phaco is the closure of the clear corneal incision (CCI), followed by intraocular pressure (IOP) management using a tactile and subjective method based on the amount of balanced salt solution injected in the anterior chamber.

The fluid influx from the ocular surface into the eye can carry microorganisms and particles into the anterior chamber after phaco, especially in hypotonic eyes^(1^-^9)^. A leaking CCI is associated with a 44-fold increased risk of endophthalmitis^(10^,^11)^. Therefore, estimating and managing the IOP after phaco is important to reduce the incidence of this sight-threatening condition.

IOP variations during the postoperative period and their optimal levels are yet not fully understood and depend on several factors. Shingleton et al.^(12)^ questioned whether the eye should be left slightly hypotonic in anticipation of a pressure spike or slight hypertension after surgery to prevent a possible vision-threatening hypotony and potential infection. Although some studies have measured IOP using Goldman applanation tonometry (30 min later)^(12^,^13)^, iCare rebound tonometry (immediately after surgery)^(14^,^15)^, and Tono-Pen (25 min after speculum removal)^(16)^ postoperatively, the accuracy of the surgeon to establish the actual IOP intraoperatively has not been evaluated.

This study aimed to compare the estimated IOP mea-sured using the subjective tactile method to the actual IOP measured by a Perkins tonometer (objective measurement) after a noncomplicated phaco. This study also compared IOP measurements obtained among surgeons with variable surgical experience.

## METHODS

This prospective study was performed at Hospital das Clínicas, Universidade de São Paulo. The study was approved by the local review board and conducted according to the Declaration of Helsinki. Informed consent was obtained from all patients before enrollment.

Eighty-one patients who underwent a noncomplicated surgery were included. Patients with corneal irregularities that prevented proper contact for IOP mea-surement, history of pars plana vitrectomy, glaucoma filtering procedures, and any anticipated difficulties with examination or analysis were excluded. There were 46 (%) females, and the mean (range) age was 71.64 (41-90) years.

Surgeons were divided into two groups. Those who had performed <120 phacos in the past 3 years were considered “less experienced surgeons” (Group 1), and those who had performed >120 phacos were considered “more experienced surgeons” (Group 2). Twenty-seven surgeons were included, of which 13 were allocated to Group 1 and 14 to Group 2; 20 were ophthalmology residents, 4 were cataract fellows, and 3 were senior surgeons. The average (range) number of previous surgeries performed was 76 (1-119) for Group 1 and 225 (120-551) for Group 2.

The estimated IOP measurement was obtained after corneal sealing after surgery while the patient was still in the surgical field with an eye speculum. The surgeon could use their usual tactile method to estimate the IOP (e.g., use of a cotton swab or an irrigation cannula). All surgeons performed their surgeries as usual.

Next, a clinical researcher in sterile clothing instilled one drop of new sterile fluorescein and used a Perkins tonometer with sterile tonometer tips (Tonosafe^®^, Haag-Streit, UK) to measure the objective IOP. The same examiner performed all IOP measurements while the patients were still in the supine position with the lid speculum opened and under topical anesthesia. The tonometer was previously tested in clinical settings and calibrated to ensure the reliability and reproducibility of IOP measurements. The tonometer was also tested in vertical and supine positions to check for orthostatic differences. To ensure no leaking caused by the researcher’s measurement, leading to a false hypotonic value, the examiner asked the surgeon to look for signs of hypotony (such as a shallow anterior chamber or wound leakage) under the microscope. The surgeon and the researcher were blinded to each other’s measurements. Hypotonic eyes were submitted to intracameral injection until proper IOP was obtained before finishing surgery.

### Statistical analysis

The sample size was calculated to achieve a statistical power of 0.8 at a significance level of 0.05, detecting a medium effect size of 0.65. Data were summarized numerically with counts and percentages and means and standard deviation. Student’s *t* test was used to compare subjective and objective IOP values, and an unpaired *t* test was used to compare subjective and objective IOP measurements between both groups of surgeons. Differences were considered statistically significant when p<0.05. An additional Bland-Altman analysis was used to evaluate the concordance of measurements among surgeons. Statistical analysis was performed using SPSS version 24 (IBM Corp.) or Stata version 18 (StataCorp LP, College Station, TX, USA).

## RESULTS

The mean objective IOP (9.14 mmHg; SD=5.86) was statistically significantly lower (p<0.001) than the mean subjective IOP (19.21 mmHg; SD=4.82).

Twenty-five eyes (30.86%) presented with a measured IOP <6 mmHg (hypotonic cases). Objective pressures between 6 and 9 mmHg were present in 23 eyes (28.40%), and pressures between 10 and 21 mmHg (normotonic) were found in 33 eyes (40.74%). No hypertonia cases (IOP >21 mmHg) were observed in this study.

The mean subjective and objective IOP measured by both groups are shown in table 1. The mean subjective IOP measurements were 18.8 mmHg (SD=5.19) and 19.5 mmHg (SD=4.46) for Groups 1 and 2, respectively. The mean objective IOP measurements were 10.32 mmHg (SD=6.65) and 7.97 mmHg (SD=4.76) for Groups 1 and 2, respectively. There was no statistical difference between the groups. Comparing the results from both groups, the difference between the mean subjective IOP (p=0.541) and objective IOP (p=0.71) was not statistically significant. However, the difference between the estimated and measured values from each group showed a statistically significant difference (p<0.001; Table 1).

**Table 1:** Results from Group 1 (less experienced surgeons) and Group 2 (more experienced surgeons)

	Group 1 (n=40 surgeries)	Group 2 (n=41 surgeries)	p-value (Group 1 vs. 2)
Mean subjective IOP	18.8 mmHg	19.5 mmHg	0.541
Mean objective IOP	10.32 mmHg	7.97 mmHg	0.71
Difference between subjective and objective IOP	8.48 mmHg (p≤0.001)	11.53 mmHg (p≤0.001)	

**Figure 1:**
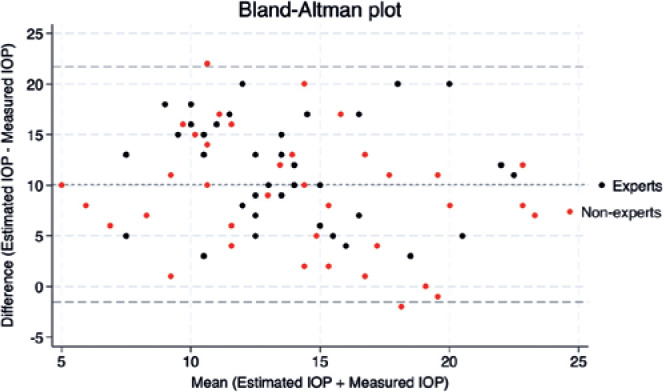
Bland-Altman plot showing agreement between IOP expert and nonexpert measurements.

An agreement between IOP measurements was evaluated by a Bland-Altman plot, as demonstrated in figure 1. The mean ± SD difference between the estimated and measured IOP was 10.1 ± 5.8 mmHg (95% limits of agreement, -1.3 to 21.5 mmHg; Figure 1). No proportional bias was present, but a fixed bias was observed across the range of IOP values upon inspection of Bland-Altman plots. There was a statistically significant relationship between the difference and mean estimated and measured IOP values (Bradley-Blackwood test, F=127.570, p<0.0001; Figure 1).

## DISCUSSION

This study evaluated the IOP after cataract surgery using two different methods. Results demonstrated that objective IOP measured by a Perkins tonometer (gold standard) was significantly lower than subjective IOP measured by tactile methods during the immediate postoperative period. This study showed that the subjective method is unreliable compared to the gold standard and does not improve with surgeons’ experience.

Hypotony (<6 mmHg) was observed in 30.86% of the eyes. Shingleton et al. also reported extreme hypotony in 20.5%^(12)^ and 6.1%^(13)^ of the patients when IOP was measured 30 min after surgery. Values could vary from this study, as some patients could have been inadvertently left with smaller pressures and were on their way to normalization 30 min later, and the results referred to a single surgeon. Although other studies indicated that IOP returns to normal levels postoperatively in 9 min^(14)^, 15 min^(15)^, or 25 min^(16)^, the effect and incidence of complications due to transitory extremes of IOP levels (hypotony or hypertonia) to ocular health are not well established.

Endophthalmitis is a rare but serious complication in cataract surgery. With the growing volume of cataract surgeries worldwide, increased endophthalmitis incidence might lead to a large absolute number of cases. Theories to explain endophthalmitis cases with sutureless CCI are usually based on the stability of the surgical wound. In a review of >22,000 cataract surgeries, Montan et al.^(17)^ reported that wound abnormality is a statistically significant risk factor for infection. Maxwell et al.^(18)^ reported that 80% of the postoperative endophthalmitis cases were related to wound defects such as leakage, wound gap, and/or malposition. Furthermore, the integrity of the corneal incision may vary according to IOP levels. Low pressure is a risk factor for endophthalmitis. Some experimental models studied the behavior of CCI under low-pressure conditions and described them as incompetent^(8^,^9^,^19^,^20)^. Behrens et al.^(6)^ analyzed cataract incisions after 24 h of an uneventful phaco and stated that corneas that presented with a gap in the CCI visualized by optical coherence tomography had a pressure of 10 mmHg (the lowest in his series). Even lid squeezing and the natural unconscious blinking cause variations in IOP in human and animal models^(21^-^23)^. Any factor that might reduce the IOP until the epithelial plug fills the external incision can lead to CCI wound incompetence. Hence, hypotony in the early postoperative period can lead to CCI incompetence, followed by intraocular infection.

However, Hayashi et al.^(14)^ reported mean IOP values of 27.6 and 29.4 mmHg in micro and small incision surgeries, respectively, and no hypotony cases in surgeries performed by a single surgeon with an IOP target between 15 and 40 mmHg. This target range can be broad and potentially driven by the inability of the surgeon to reach the approximate pressure he desires in each surgery. Their results suggested that when IOP is adjusted to normal or relatively high with stromal hydration, it will not cause wound outflow, resulting in hypotony, supporting the idea of a preferable higher IOP by the end of the surgery.

No significant difference was found in measuring subjective IOP between more and less experienced surgeons. Because the ability to determine the IOP is subjective, it is probably unrelated or improves over time. There is no learning curve for this surgical step, as no objective measurements are performed to check the final IOP.

Analyzing each surgeon separately, a pattern was observed among most surgeons: the average difference between subjective and objective IOP was similar in all surgeries. This finding suggested that subjective IOP measures might be reproducible, although not necessarily accurate.

A limitation of this study is that it was conducted in one center (academic residency program), and results will probably vary among different residency programs. Nevertheless, there are no training models that objectively prepare residents for this surgical step. Some residency programs use virtual reality in association with formal lectures, wet labs, and live surgical experiences to train their residents^(24)^. However, surgical simulators do not include IOP target training. The management of the final IOP mostly depends on the senior surgeon’s experience to demonstrate it. As there were no differences in measuring the IOP between experienced and inexperienced surgeons, an objective training model should be developed.

In conclusion, subjective IOP measured by the tactile surgeon method differed significantly from objective IOP measured by a Perkins tonometer, suggesting that tactile IOP measurements can be inconsistent and inaccurate. Nevertheless, establishing standard IOP assessment methods by the end of the surgery and providing appropriate resident training are paramount to improve surgeons’ ability to determine IOP assessment after cataract surgery.
